# Effect of Acrylamide on Oocyte Nuclear Maturation and Cumulus Cells Apoptosis in Mouse *In Vitro*

**DOI:** 10.1371/journal.pone.0135818

**Published:** 2015-08-14

**Authors:** Shuzhen Liu, Ligang Jiang, Tao Zhong, Shuhui Kong, Rongbin Zheng, Fengyun Kong, Cong Zhang, Lei Zhang, Liguo An

**Affiliations:** 1 Key Laboratory of Animal Resistance Research, College of Life Science, Shandong Normal University, Jinan, China; 2 Center for Reproductive Medicine, Qilu Hospital of Shandong University, Jinan, China; 3 Reproductive Medicine Center, the Second Hospital Affiliated to Shandong University of Traditional Chinese Medicine, Jinan, Shandong, China; Nanjing Agricultural University, CHINA

## Abstract

Acrylamide (ACR) is a chemical compound with severe neurotoxicity, genotoxicity, carcinogenicity and reproductive toxicity. Recent studies showed that ACR impairs the function of reproductive organs, e.g., epididymis and testes. *In vitro* maturation of mouse oocyte is a sensitive assay to identify potential chemical hazard to female fertility. The aim of this study was to evaluate the adverse effects of ACR on the nuclear maturation and cumulus cells apoptosis of mouse oocytes *in vitro*. Cumulus–oocyte complexes were incubated in a maturation medium containing 0, 5, 10 and 20 μM of ACR. Chromosome alignment and spindle morphology of oocytes was determined by immunofluorescence and confocal microscopy. Our results showed that oocytes exposed to different doses of ACR *in vitro* were associated with a significant decrease of oocyte maturation, significant increase of chromosome misalignment rate, occurrence of abnormal spindle configurations, and the inhibition of oocyte parthenogenetic activation. Furthermore, apoptosis of cumulus cells was determined by TUNEL and CASPASE-3 assay. Results showed that apoptosis in cumulus cells was enhanced and the expression of CASPASE-3 was increased after cumulus–oocyte complexes were exposed to ACR. Therefore, ACR may affect the nuclear maturation of oocytes via the apoptosis of cumulus cells *in vitro*.

## Introduction

Acrylamide (ACR) is a water-soluble vinyl monomer used to produce polymers and gels. ACR is widely used in industry, science and technology, e.g., water purification, paper and fabric manufacturing, the mining, and gel electrophoresis. However, occupational exposure to the burgeoning industrial use of ACR could potentially lead to neurotoxicity that may induce skeletal muscle weakness, numbness of the extremities, ataxia, and cognitive impairment [1, 2]. Moreover, many studies using laboratory animals suggest that the ACR-induced neurotoxicity is similar to the neurological symptoms observed in human intoxication [3, 4]. In addition to occupational exposure, food is also one of the main sources of ACR uptake. For example, significantly higher level of ACR was found in a variety of cooked foods produced by a Swedish company [5].

Exposure to monomeric ACR has been shown to result in peripheral neuropathy, occurrence of tumor, gene mutations, and reproductive lesion. Treatment of PC12 cells with ACR induces apoptosis through oxidative stress of ROS production [6]. Moreover, ACR can increase the incidence of certain tumors, such as bladder cancer in human mammary gland, fibroadenomas in female rats, and tunica vaginalis mesotheliomas in male rats [7, 8]. Studies on rodent have also indicated that ACR increases DNA strand breaks, the rate of spontaneous abortion, neonatal mortality, and results in a decrease in the total number of the offspring in rodents [9, 10]. ACR also increases the gene mutations in germ cells, which results in clastogenic and aneuploidy effects [11, 12]. Extensive studies have focused on the neurotoxicity and carcinogenicity of ACR. Yet, the effects of ACR on female reproductive toxicology and embryo toxicity were not fully understood.

Oocyte maturation is a process of nuclear maturation accompanied with a series of physiological changes of the ooplasm, e.g. germinal vesicle breakdown and extrusion of polar body, and is vital to successful fertilization and subsequent embryo development [13, 14]. Nuclear maturation is regard as a physiological incident that the developing oocyte departs from meiotic Prophase-I and arrests at Metaphase-II (MII) [15]. During nuclear maturation of oocyte, both precise chromosome migration and proper condensation and separation are key steps. In the phase of MII, chromosomes are concentrated, aligned at Metaphase Plate and do not oscillate [16]. Recently, many studies were focused on toxin-induced effect on oocyte nuclear maturation. For example, Lenie et al. [17] found that polar body extrusion of mouse oocyte was decreased by 30 μM bisphenol A, implying that oocyte maturation was suppressed. Grossman et al. [18] reported that the proportion of bovine oocytes of nuclear maturation was reduced after exposure to 50 μM mono-(2-ethylhexyl) phthalate (MEHP). Furthermore, 2-Methoxyestradiol, an endogenous metabolite of 17beta-estradiol which may be increased after environmental pollutants exposure, significantly inhibited nuclear maturation of mouse oocytes and induced chromosome congression failure [19]. Zenzes and Bielecki [20] reported that mouse oocytes number entries into MII-stage were decreased and chromosomes were dispersed after exposure to 10 mmol/L nicotine for 8 h. Furthermore, it has been reported recently that oocyte quality and fertility of mouse were reduced by feeding with 10 or 50 mg/kg/d ACR, as shown by increased percentage of GV-stage oocyte, an aberrant cytoskeletons (an increased abnormal spindle rate), ROS generation, apoptosis induction, and changes of epigenetic modifications in oocytes [21]. However, reports about effects of ACR on nuclear maturation of oocyte were not been investigated fully, especially the influences of ACR on chromosome in oocytes.

Cumulus cells surround oocyte and provide crucial nutrients and signals. Cumulus cells are essential for oocyte maturation, including the process of meiotic arrest and resumption [22]. Moreover, it has been demonstrated that cumulus cells affect chromatin condensation of mouse oocyte *in vitro* [23]. Pocar et al. found that apoptotic cumulus cell were increased significantly and oocyte maturation was decreased after bovine COCs were cultured with environmental toxin (polychlorinated biphenyls) *in vitro* [24]. Our previous study showed that apoptosis occurred in the cumulus cells which surrounded the ovulated oocyte after the mouse was treated with polychlorinated biphenyls [25]. Furthermore, Wei et al. found that viability of granulosa cells was decreased after exposure to 0.5 and 5.0 mM ACR *in vitro* [26]. However, no reports were available regarding to the influence of ACR on cumulus cells, especially the apoptosis in cumulus cells.

In this study, the effects of ACR on the oocyte nuclear maturation, apoptosis of cumulus cells, and the potential development of mouse oocytes *in vitro* were evaluated. These results will provide the important reference for the understanding and prevention of reproductive disorders caused by ACR.

## Materials and Methods

### Animals and chemicals

Animal care and use were conducted in accordance with the Animal Research Institute Committee guidelines of the Ethics Committee of Shandong Normal University, China. Mice were housed in a temperature-controlled room with proper darkness—light cycles, fed with a regular diet, and maintained under the care of the Laboratory Animal Unit, Shandong Normal University, China. Mice were euthanized by cervical dislocation. This study was approved by the Committee of Animal Research Institute, Shandong Normal University, China.

Female ICR mice, 5–7 weeks old, were provided by the Beijing HFK Bio-Technology Co. Ltd (Beijing, China). Animals were kept in plastic cages and given free access to food and water. Mice were allowed at least 1 week to adapt for the experimental conditions and were randomly assigned into four groups. The oocytes from these four groups were treated with 0, 5, 10, 20 μM of ACR, respectively.

Pregnant mare serum gonadotropin (PMSG) was purchased from the Ningbo Second Hormone Factory (China). Chemicals used in the present study were obtained from Sigma (St. Louis, MO) unless otherwise noted.

### Collection and maturation of oocytes

The mice were superovulated with an intraperitoneal injection of 10 IU PMSG and then sacrificed via cervical dislocation 44–46 h later. The cumulus—oocyte complexes (COCs) were collected by breaking the intumescent ovarian follicles. After three times of washing in M2 medium, the COCs were cultured in a prepared M2 media supplemented with 0, 5, 10, 20 μM of ACR at 37°C in a humidified atmosphere with 5% CO_2_ for 14 hours. The *in vivo* maturation (IVM) rates were determined by the presence of the first polar body (PB I). In this study, only oocytes with first polar bodies were used for activation assay and morphological analysis of the spindle.

### *In vitro* fertilization

After IVM, cumulus cells were removed by 0.1% hyaluronidase added to the maturation medium. Denuded oocytes were washed twice in maturation medium and three times in G-IVF (fertilization medium, Vitrolife). Subsequently, denuded oocytes were fertilized with fresh cauda epididymal sperms (1×10^6^/ml motile, obtained from an ICR male donor) that were pre-incubated in G-IVF for 1 h under a steady temperature (37°C) and humidity condition. Gametes were co-incubated in 50 μL G-1 medium covered with mineral oil for 6 hours at 37°C with 5% CO_2_ in air. The presence of two polar bodies and pronuclei were used as the criteria of successful fertilization. Results were observed at 1 day for 2-cell.

### Immunofluorescence staining and confocal microscopy

After culturing *in vitro* for 14 hours, oocytes were fixed with 4% (w/v) paraformaldehyde in PBS (pH 7.4) for 40 min at room temperature. Fixed samples were permeabilized in the incubation buffer (0.5% Triton X-100 in 20 mM Hepes, pH 7.4, 3 mM MgCl_2_, 50 mM NaCl, and 300 mM sucrose) for 30 min. Following two times of washing with PBS containing 0.01% Triton-X100, the samples were blocked in PBS containing 1% BSA for 1 hour at RT. The oocytes were subsequently incubated with a fluorescein isothiocyanate-labeled mouse monoclonal antibody against α-tubulin (show spindle of oocyte) or CASPASE-3 (abcam) diluted in 1% BSA (1:100) for 1 hour at 37°C to visualize the microtubules. Chromatin was stained with DAPI in PBS (1:100) for 10 min. Finally, the samples were examined under a Leica confocal laser scanning microscope.

### Assessment of oocyte activation

Oocytes were incubated in the Ca^2+^-free CZB activating medium supplemented with 10 mM SrCl_2_ at 37°C in a humidified atmosphere with 5% CO_2_ in air before being observed under a microscope. Oocytes with one (1PN) or two pronuclei (2PN), or two cells each having a nucleus (2-cell) were considered as being activated.

### TUNEL assay

After exposure to ACR, COCs were fixed in 4% (v/v) paraformaldehyde solution for 1 hour and permeabilized in 0.1% Triton X-100 in 0.1% citrate solution for 1 hour at room temperature. Subsequently, COCs were incubated in TUNEL reaction medium (Roche, Mannheim, Germany) for 1 hour at 37°C in the dark. After the reaction was stopped, COCs were washed three times in PBS and the total cell nuclei were labeled with 10 mg/ml Hoechst for 5 min in the dark. The slides were observed by confocal laser scanning microscopy. Cells with fragmented nuclei stained in red by TUNEL reaction medium are considered as apoptotic cells.

### Statistical analysis

All data were analyzed using SPSS 18.0 statistical software. At least three replicates were conducted for each treatment. The data were expressed as means ± SD and analyzed using chi-square and student *t* tests. Significant differences were defined as *P*< 0.05.

## Results

### Effect of ACR on the maturation of oocytes *in vitro*

**Fig 1:**
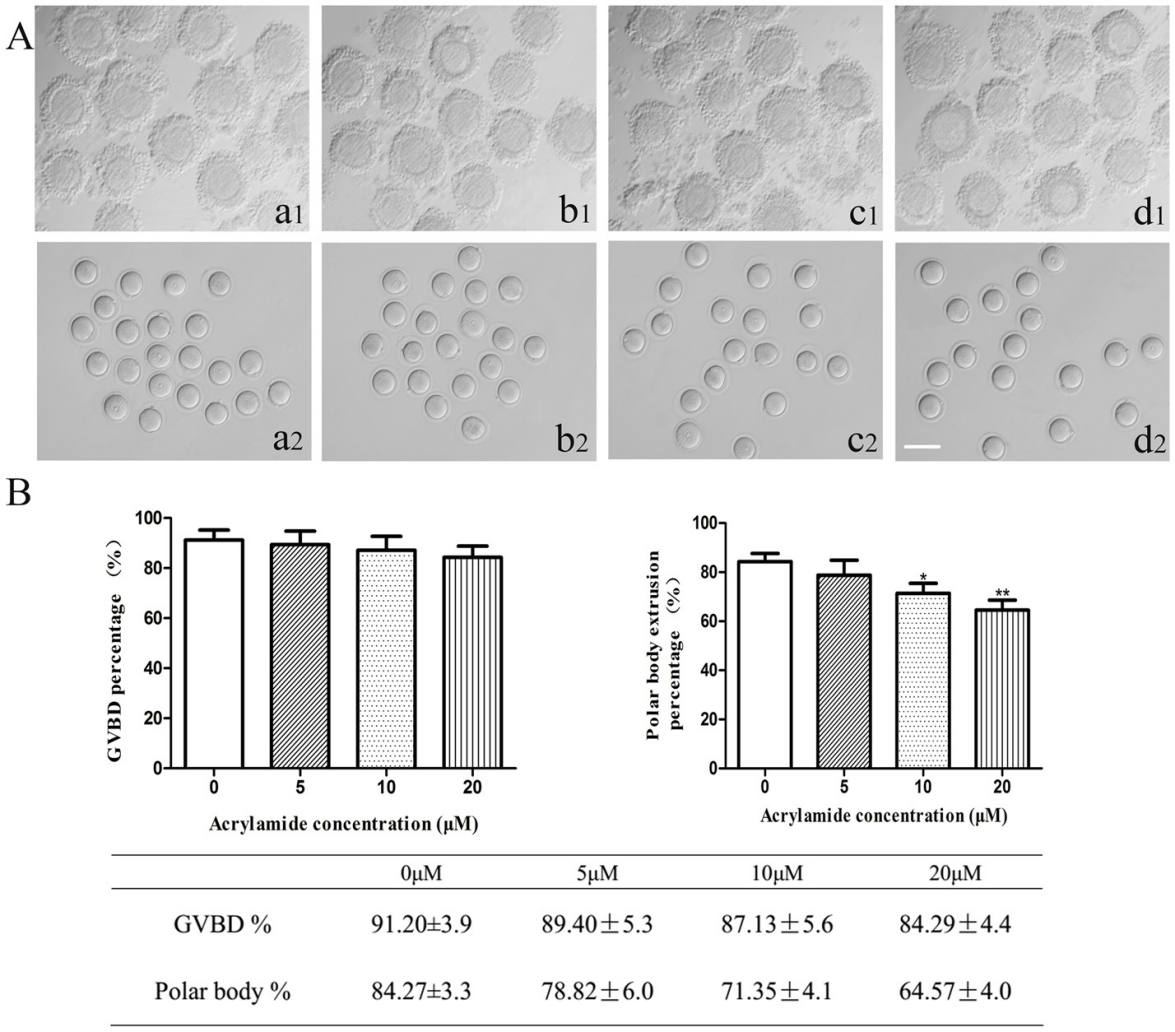
Effect of ACR on the maturation of mouse oocytes (COCs) *in vitro*. (A) COCs morphology before culturing (a1, b1, c1, d1) and the polar body I (PB I) of oocyte which cumulus cells were removed after COCs cultured for 14h treated with acrylamide of 0 μM (a2), 5 μM (b2), 10 μM (c2) and 20 μM (d2); Bar = 150 μm. (B) The percentage of GVBD and PB I after COCs cultured for 14h treated with experimental dosage of acrylamide. The data was compared using chi-square test and *t*-tests. Data is expressed as the mean ± SD of three separate experiments. * indicates *P* < 0.05 vs. control, ** indicates *P*<0.01 vs. Control. 0 μM group: n = 147; 5 μM group: n = 164; 10 μM group: n = 172; 20 μM group: n = 168.

With increasing concentration of ACR (0, 5, 10 and 20 μM), percentage of GVBD of oocytes was not affected when compared to the untreated oocytes (*P* > 0.05) (Fig 1B). However, the percentage of polar body I extrusion in oocytes treated with 10 or 20 μM ACR was significantly lower than that of untreated control (84.27 ± 3.3%, *P* < 0.05) (Fig 1B).The rate of polar body I extrusion after oocytes being exposed to 10 μM and 20 μM ACR was 71.35 ± 4.1% and 64.57 ± 4.0%, respectively. Treatment with 5 μM ACR reduced the percentage of polar body I extrusion to 78.82 ± 6.0%, but no significant difference was observed when compared to the untreated control (*P* > 0.05).

### Effect of ACR on parthenogenic activation of oocyte

**Fig 2:**
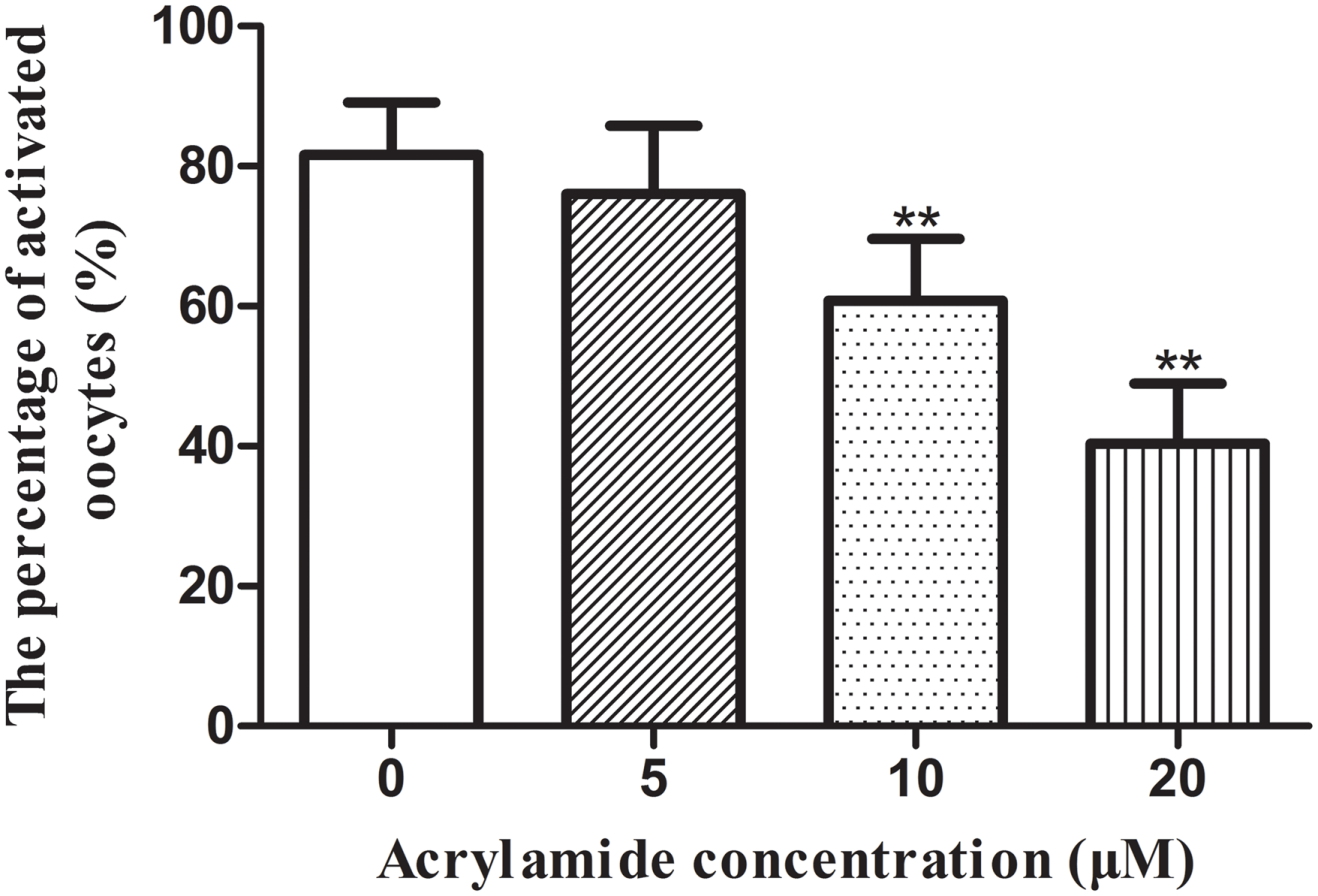
Effect of ACR on the parthenogenetic activation of oocytes. Data are expressed as the mean ± SD of three separate experiments. ** indicates *P* < 0.01 vs control. 0 μM group: n = 135; 5 μM group: n = 140; 10 μM group: n = 138; 20 μM group: n = 143.

The parthenogenetic activation rate of oocytes in 0, 5, 10 and 20 μM ACR-treated groups was 81.63 ± 7.5%, 76.03 ± 9.7%, 60.80 ± 8.8% and 40.37 ± 8.5%, respectively. There were significant reductions of parthenogenetic activation rate in the 10 μM and 20 μM groups (*P* < 0.05), but not in the 5 μM group compared to the untreated control (Fig 2).

### Effect of ACR on the chromosome and spindle of oocytes after IVM for 14 h

**Fig 3:**
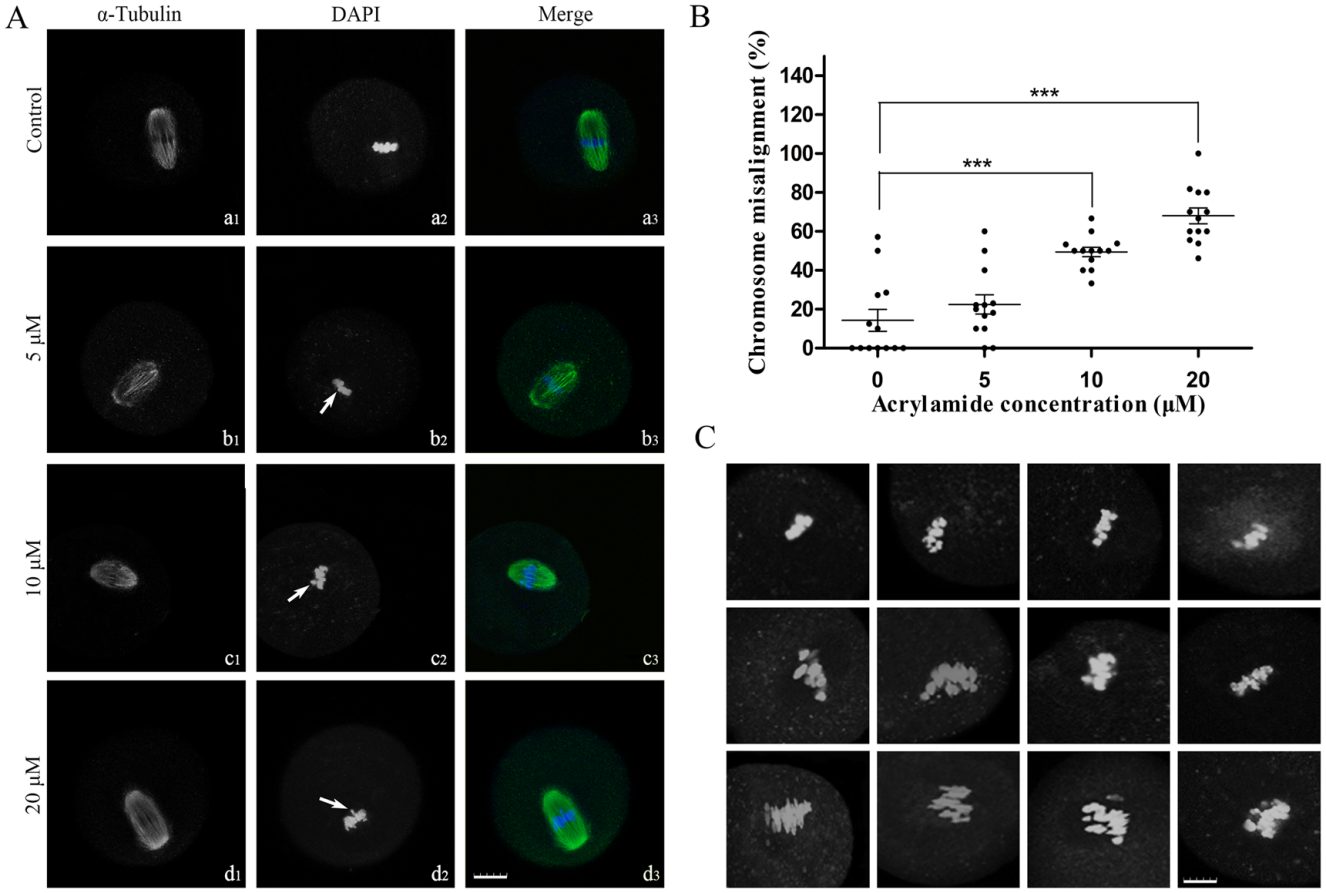
Effect of ACR on the chromosome and spindle of oocytes after ACR treated for 14h. After COCs were treated with ACR for 14h, cumulus cells were removed and the morphology of spindle and chromosomes configuration of oocytes was observed. (A) Representative confocal micrographs of spindle and chromosomes. Spindle morphology of oocytes was displayed in a1, b1, c1, d1 and nuclei was displayed in a2, b2, c2, d2. a3, b3, c3, d3 is the merge of (a1, a2), (b1, b2), (c1, c2), (d1, d2), respectively; Chromosome of oocytes was aligned (a2) in control group and misalignment (b2, c2, d2) in ACR-treated group. Arrows label the misalignment chromosomes. (B) The percentage of oocytes of chromosome misalignment in different group. Each dot in the coordinate represents one experimental data, and each group was 13 replicates. The percentage of chromosome misalignment = the number of chromosome misalignment oocytes / (the number of chromosome alignment oocytes and the number of chromosome misalignment oocytes).The data was compared using chi-square test and t-tests. Data are expressed as the mean ± SD. *** indicates *P* < 0.0001 vs. Control and means significant difference between experiment group and control group. (C) Images for various chromosome misalignment. 0 μM group: n = 110; 5 μM group: n = 139; 10 μM group: n = 146; 20 μM group: n = 137. Bar = 25 μm.

The chromosomes of ACR-treated oocytes at MII-stage were misalignment, while the chromosome in the control oocyte was aligned normally (Fig 3A). Meanwhile, the abnormal spindle morphology was observed in the ACR-treated oocytes (Fig 3A). The percentage of oocytes of chromosome misalignment in 0, 5, 10 and 20 μM ACR-treated groups was 14.27 ± 5.6%, 22.49 ± 4.9%, 49.43 ± 2.4% and 68.00 ± 4.0%, respectively. The percentage of oocytes with misaligned chromosome in the treatments of 10 and 20 μM ACR was significant higher than those in the control.

### Effect of ACR on the rate of fertilization and cleavage of embryo *in vitro*

After the COCs were cultured with exposure to ACR *in vitro* for 14 h, then denuded oocytes were get and *in vitro* fertilization the capacity of developing to 2-cell from zygote were performed. The data was shown in Table 1. There were no significant effects of fertilization rate, 2-cell rate in the group of 5 μM ACR compared to the control (*P* > 0.05). However, the fertilization rate, 2-cell rate in 10 μM and 20 μM ACR-treated groups were significantly lower than those of the untreated oocytes (*P* < 0.05).

**Table 1:** The effects of ACR treatment on mouse fertilization and preimplantation embryonic development.

Group (μM)	Total number of oocytes	Fertilization (%)	2-cell (%)
0	147	127 (86.6 ± 1.2)	102 (80.6 ± 0.9)
5	164	126 (76.9 ± 8.1)	94 (74.6 ± 11.3)
10	172	115 (67.2 ± 8.6) *	73 (64.1 ± 11.8)*
20	168	91 (54.3 ± 12.9) **	43 (47.5 ± 15.6)**

Data are expressed as the mean ± SD of three separate experiments.

* *P*<0.05 vs. control,

** *P*<0.01 vs. control.

### Effect of ACR on apoptosis of cumulus cells

**Fig 4:**
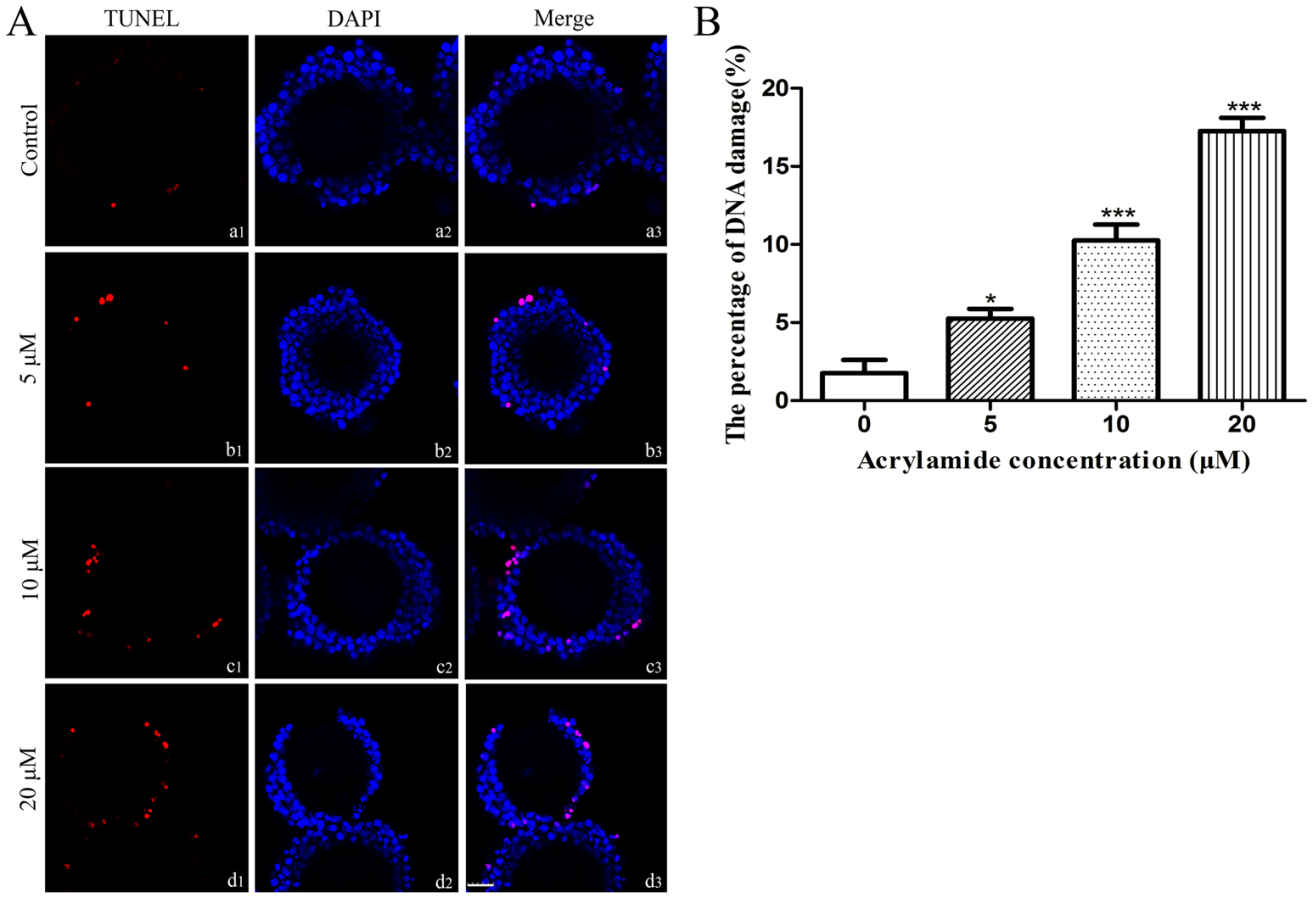
Representative TUNEL assay images of COCs after exposure to ACR. (A) COCs were treated with 0 μM (a1, a2, a3), 5 μM (b1, b2, b3), 10 μM (c1, c2, c3) and 20 μM (d1, d2, d3) of ACR. Apoptotic cells are displayed in red (a1, b1, c1, d1) and nucleuses are displayed in blue (a2, b2, c2, d2). a3, b3, c3, d3 is the merge of (a1, a2), (b1, b2), (c1, c2), (d1, d2), respectively. Bar = 25 μm. (B) The percentage of TUNEL positive cumulus cells in 0, 5, 10 and 20 μM ACR-treated group. Data is expressed as the mean ± SD of three separate experiments. * indicates P < 0.05 vs. control, *** indicates P < 0.001 vs. Control.

In the control group, few apoptotic cumulus cells (red) were observed after COCs were cultured for 14 h. In contrast, more apoptotic cumulus cells were observed in the 5, 10 and 20 μM ACR-treated groups than those in the control group (Fig 4A). The percentages of positive cumulus cells in the 0, 5, 10 and 20 μM ACR-treated groups were 1.75 ± 0.9%, 5.25 ± 0.6%, 10.25 ± 1.0%, 17.25 ± 0.9%, respectively. The percentages of TUNEL positive cumulus cells in the groups of 5, 10, 20 μM ACR were significantly higher than those in the control group (Fig 4B). Furthermore, the work that checking oocytes’ DNA damage by TUNEL assay after culturing COCs 14h in ACR was performed. The results indicate that there is no breakage in DNA strand in oocytes (S1 Fig).

**Fig 5:**
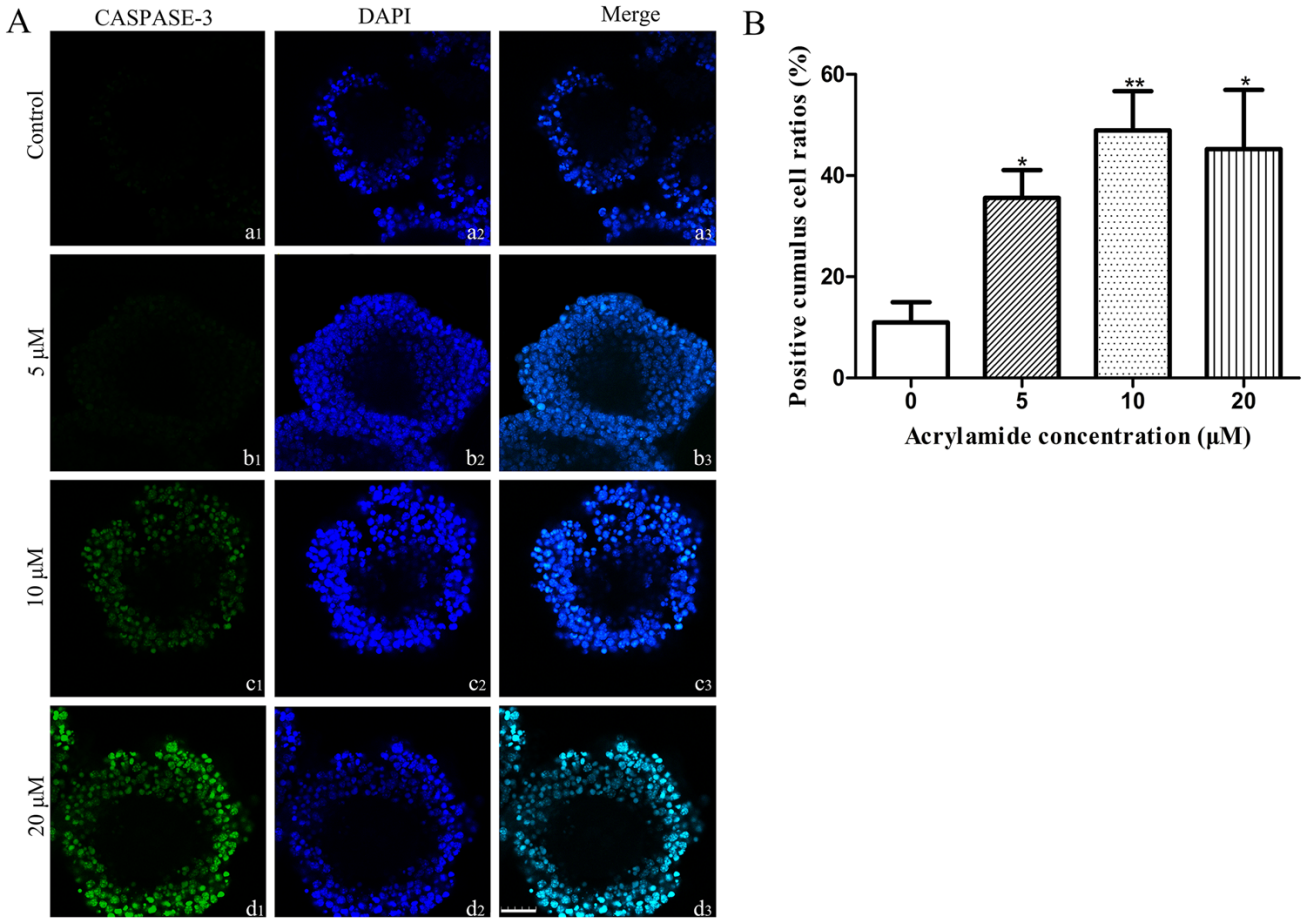
Detection of CASPASE-3 in cumulus cells by immunofluorescence after COCs were cultured with acrylamide for 14h. (A) COCs representative samples from the control group (a1, a2 and a3), the 5 μM acrylamide-treated group (b1, b2 and b3), the 10 μM acrylamide-treated group (c1, c2 and c3), and the 20 μM acrylamide-treated group (d1, d2 and d3) are shown. Green staining indicates CASPASE-3 expression in cumulus cells (a1, b1, c1, d1), cell nucleus are stained blue (a2, b2, c2, d2), a3, b3, c3, d3 is the merge of (a1, a2), (b1, b2), (c1, c2) and (d1, d2), respectively. Bar = 25 μm. (B) Positive cumulus cell ratio of CASPASE-3 protein in 0, 5, 10 and 20 μM ACR-treated group. The number of positive cumulus cell was counted and the ratio to the number of nuclei was analyzed for each group. Data is expressed as the mean ± SD of three separate experiments. * indicates *P* < 0.05 vs. control, ** indicates *P* < 0.01 vs. Control.

The immunofluorescence of CASPASE-3 in cumulus cell treated with 5 μM ACR were increased compared to the control group, and the immunofluorescence of CASPASE-3 in cumulus cell treated with 10 and 20 μM ACR were significantly higher than that in the control group (Fig 5A). Furthermore, positive cumulus cells ratios of CASPASE-3 in 0, 5, 10 and 20 μM ACR-treated group were 10.99 ± 4.0%, 36.58 ± 5.5%, 48.91 ± 7.8%, 45.20 ± 11.7%, respectively. The positive cumulus cells ratios of CASPASE-3 in 5, 10 and 20 μM ACR-treated group were significantly increased compared to those in the control group (Fig 5B).

## Discussion

In 2002, studies showed that a variety of food products contained high level of ACR [27]. Therefore, it became important to determine the effect of ACR on health of humans. As a result of interactions between the aminoacid asparagine and sugars, ACR can be generated from starchy food products during heating process at temperatures above 100°C [28]. For example, high level of ACR (2300 μg/kg) has been observed in potato chips and French fries [29], suggesting that diet is one of main sources of environmental ACR exposure for humans [30]. ACR can be readily absorbed through the oral cavity [31]. Moreover, it has been demonstrated that ACR can also be absorbed through the skin [32]. In addition, ACR has been shown to be toxic to the reproductive system [9, 33–35].

Relatively few studies have been conducted to investigate the effect of ACR on mouse oocytes. In this study, cumulus-oocyte-complexs (COCs) were utilized to investigate the ACR toxicity on oocyte *in vitro*. The existence of cumulus cells may make the study more complex when investigating the effect of ACR on oocytes, but naturality and facticity is more important. An antral follicle contains an arrested oocyte and multideck granulosa cells which are around with oocyte. Cumulus cell belongs to one of the granulosa cell, thus, using COCs to conduct a *in vitro* toxicological study may be better to simulate the reality, and also help come up with a hypothesis that ACR may affact the oocyes via impairing the cumulus cells. Furthermore, it has been proved that mice [36], bovine [37] and porcine [38] culturing COCs *in vitro* is beneficial for the oocyte maturation than those culturing denuded oocytes only.

Results of this study showed that ACR inhibited the PB I extraction (Fig 1B) and increased the percentage of chromosome misalignment in MII-stage mouse oocytes (Fig 3B) in a dose-dependent manner. In this study, these results indicate that nuclear maturation of oocyte was incomplete after exposure to ACR *in vitro*. Meanwhile, abnormal meiotic spindle morphology could also be found in the chromosome misalignment oocyte after COCs being exposed to experimental dosage of ACR for 14 h (Fig 3). Alignment of chromosomes in phase of metaphase and then proper segmentation of individual chromosomes in the transition from metaphase to anaphase is essential to maintain the genomic stability of the gametes. During meiosis I and II, meiotic spindles are vital for the separation of maternal chromosomes [39]. Spindles have been shown to be sensitive to xenobiotic chemicals, temperature [40] and drugs [41]. Recently, spindle analysis was utilized to assess the oocyte quality [42] and the impacts of drugs and toxicants on oocytes [43, 44]. It has been reported that MAPK and MPF may have important effects on the regulation of microtubule dynamics *in vitro* [45, 46]. Furthermore, it is usually considered that chromosomes of oocyte are captured to spindle by special mechanisms, but not the spindle itself, because the length of spindle is much shorter than the diameter of germinal vesicle in the first meiosis [47]. It has been reported in starfish oocytes that a contractile actin meshwork is the special mechanism that collects chromosomes and achieves chromosome congression [48]. Therefore, ACR-induced defective nuclear maturation of oocyte may be due to the abnormal spindle, leading to chromosomes separation failure after metaphase-I, or due to the failure of chromosomes collecting by the special mechanism as mentioned above. Moreover, the defective alignment of chromosome at MII-stage may lead to the subsequent low rate of fertilization, 2-cell and parthenogenetic activation.

Parthenogenetic activation, initially described by Owen in 1849, is a process that an embryo can be developed from oocytes without spermatozoa [49]. When oocytes are activated, there is a resultant train of Ca^2+^ oscillations [50]. In this study, a medium containing strontium chloride (SrCl_2_) was employed to induce intracellular Ca^2+^ release and a series of Ca^2+^-dependent biological responses, which ultimately results in the parthenogenetic activation. Our results showed that ACR treatment significantly reduced SrCl_2_-induced parthenogenetic activation (Fig 2), suggesting that ACR may affect the intracellular Ca^2+^ release or alter the Ca^2+^ concentration in oocytes.

It is known that the surrounding cumulus cells play a crucial role in oocyte growth, maturation, and fertilization by providing nutrients and releasing and mediating signals to the oocytes [51, 52]. For instance, cumulus cells can arrest oocyte meiosis via generating oocyte maturation inhibitors (OMI) [53] and induce meiosis via mediating LH stimulation to the oocyte [52, 54]. In addition, cumulus cells have also been implicated in reducing the damage of reactive oxygen species (ROS) on oocytes by enhancing glutathione (GSH) content in oocytes [55], and probably also have an effect on attracting, trapping, and selecting spermatozoa during fertilization [52]. Cumulus cells perform these regulatory functions mainly by means of gap junctions [52] and partially by cumulus secretions, such as OMI [53], progesterone [56], inhibin [57] and activin [58]. It has been suggested that the communication between oocytes and their surrounding cumulus cells is bidirectional [59, 60], which becomes essential for oocytes growth, maturation, fertilization, and even the early development of the embryo.

Our studies showed that ACR induced apoptosis in cumulus cells as demonstrated by TUNEL assay (Fig 4). The protein of CASPASE-3 (considered as an early marker of cell apoptosis [61]) was significantly activated by both 10 and 20 μM of ACR (Fig 5). It has been reported that ACR exposure also induces apoptosis in other types of cell. For example, Guan et al. found that apoptosis of murine hepatic stem cells was induced and *Caspase-3* was upregulated by 3.5 mg/L ACR *in vitro* [62]. The level of apoptosis in the cumulus cells after maturation is negatively correlated with the development potential of the oocyte [63]. The quality of the oocyte and cumulus cells is considered as a potential factor correlated with the competence of oocytes to resume meiosis, mature, and fertilization and subsequent development *in vitro*. Thus, the occurrence of apoptosis in the cumulus cells can be used to indicate the quality of the oocyte [64]. Given the fact that bidirectional communication between cumulus cells and oocytes via gap junctions determines the cytoplasmic maturation of oocytes [60] and high degree of apoptosis in cumulus cells contributes to poor embryo development [65], the incompetence of nuclear maturation of oocytes and high degree of cumulus cell apoptosis induced by ACR may be responsible for the low fertilization rate and poor developmental capability of oocytes. Further studies will be directed to investigate the molecular mechanisms of ACR-induced cumulus cell apoptosis.

In conclusion, the results of the present study indicate that low dose of ACR induces chromosome misalignment and abnormal spindle configuration, increases the presence of apoptosis in cumulus cells, and decreases the percentage of maturation, fertilization and parthenogenetic activation of oocytes *in vitro*. Although the mechanism is still unclear, the observed disorder in chromosome congression and spindle configuration and apoptosis in cumulus cells are likely to account for the poor developmental competence of oocytes treated with ACR.

## Supporting Information

S1 Fig The effect of ACR on oocytes’ DNA damage. After culturing COCs in ACR for 14h, cumulus cells were removed and oocytes were detected. COCs were treated with 0 μM (a1, a2), 5 μM (b1, b2), 10 μM (c1, c2) and 20 μM (d1, d2) of ACR, respectively. Chromosome morphology (blue) of oocytes was displayed in a1, b1, c1, d1 and DNA breakage strand (red) displayed in a2, b2, c2, d2. The results indicate that there is no breakage in DNA strand in oocytes. 0 μM group: n = 44; 5 μM group: n = 50; 10 μM group: n = 46; 20 μM group: n = 50. Bar = 25 μm.
(TIF)
