# Supplementary figures and images for: Effect of Acrylamide on Oocyte Nuclear Maturation and Cumulus Cells Apoptosis in Mouse In Vitro
Source: PLoS One. 2015 Aug 14;10(8):e0135818. doi: 10.1371/journal.pone.0135818 (PMC4537141; doi:10.1371/journal.pone.0135818)

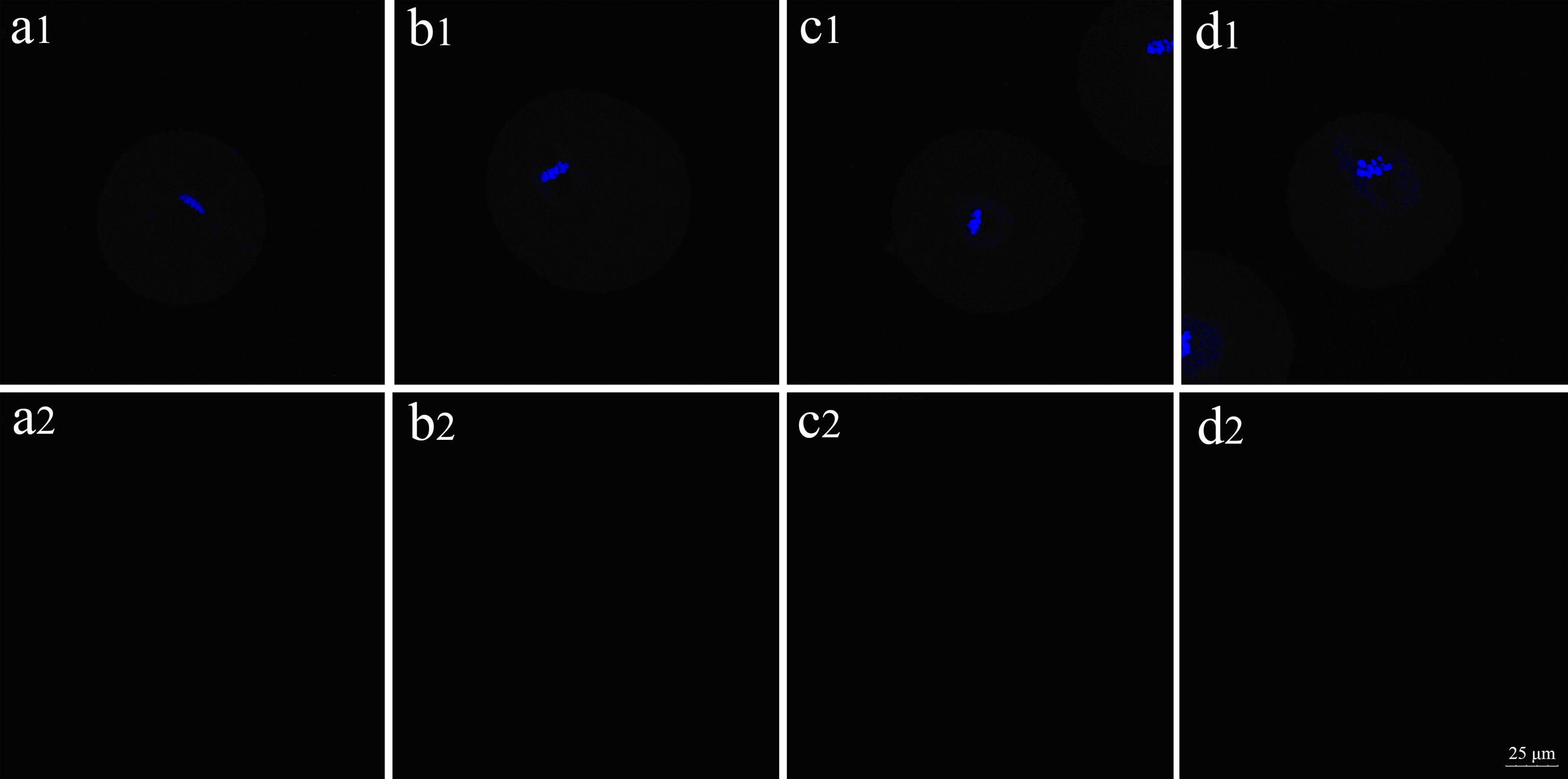

Supplement: S1 Fig — After culturing COCs in ACR for 14h, cumulus cells were removed and oocytes were detected. COCs were treated with 0 μM (a1, a2), 5 μM (b1, b2), 10 μM (c1, c2) and 20 μM (d1, d2) of ACR, respectively. Chromosome morphology (blue) of oocytes was displayed in a1, b1, c1, d1 and DNA breakage strand (red) displayed in a2, b2, c2, d2. The results indicate that there is no breakage in DNA strand in oocytes. 0 μM group: n = 44; 5 μM group: n = 50; 10 μM group: n = 46; 20 μM group: n = 50. Bar = 25 μm. (TIF) [file pone.0135818.s001.tif]
